# Phenology and pollinating wasp dynamics of *Ficus microcarpa* L.f.: adaptation to seasonality

**DOI:** 10.1186/1999-3110-54-11

**Published:** 2013-08-21

**Authors:** Hui-Wen Yang, Hsy-Yu Tzeng, Lien-Siang Chou

**Affiliations:** 1 Institute of Ecology and Evolutionary Biology, National Taiwan University, No. 1, Sec. 4, Roosevelt Rd.,, Taipei, 10617 Taiwan; 2 Department of Forestry, National Chung Hsing University, No. 250 Kuokwang Rd.,, Taichung, 40227 Taiwan

**Keywords:** *Eupristina verticillata*, *Ficus microcarpa*, Fig wasp, Insect population dynamics, Invasive plant, Mutualism, Phenology, Pollination ecology

## Abstract

**Background:**

In the obligate plant/pollinator mutualism, pollinator abundance is conditioned by the host resource. In order to investigate the population fluctuation of pollinating wasps and the phenological processes involved, this study examined the dual dynamics of the pollinator and the syconium phenology of a seasonal fruited fig tree population, *Ficus microparpa,* in Taipei, Taiwan.

**Results:**

Our results revealed three seasons in the annual phenology: spring crop, summer-fall crop and winter trough seasons. The syconium quantity was correlated most significantly with temperature based on the generalized linear model with the meteorological data transformed by a principal component analysis. The pollinator population showed an increasing trend in spring, reached the maximum abundance in summer, and then declined drastically in winter, consistent with the syconium production fluctuation. With the small amount of local pollinators from the winter syconia and potential immigrating foundresses from other populations, the pollinator population size can increase very quickly from almost zero to over 40,000 wasps for this 29-tree local population within a season.

**Conclusion:**

This syconium phenological scheme, coupled with the fast-recovery rate of pollinators, may explain the worldwide adaptation and invasion of *Ficus microcarpa.*

**Electronic supplementary material:**

The online version of this article (doi:10.1186/1999-3110-54-11) contains supplementary material, which is available to authorized users.

## Background

Pollination success is the main determinant of the fitness of the plant kingdom. Vegetal species pollinated by insects have developed numerous strategies to attract insects and transport their pollen to conspecific individuals, driving the evolution of angiosperms (Hu et al. 2008; Thien et al. 2009). However, variations in the schemes of host phenologies may result in pollination failure (Winder and Schindler 2004; Durant et al. 2007; Jonzen et al. 2007; Martin 2007). The generalist pollinating species would be less affected by these changes for they can visit a greater range of flowering plants than more specialized pollinators. More specifically, extreme specialists, such as the obligate mutualistic species, are bound to their hosts. In these cases, both species require the other for survival and to accomplish their life cycle. Changes in the abundance of any of the partners could drastically reduce their fitness (Visser and Both 2005).

The genus *Ficus* exhibits one of the most extreme obligate nursery mutualisms, with plant pollination performed by Hymenopteran wasps (Agaonidae *sensu* Cruaud et al. 2010). The characteristic shared by the *Ficus* trees is an enclosed urn-shaped inflorescence called the syconium (also known as a fig), with dozens to thousands of unisexual flowers inside. The only entrance to the syconium lumen is a narrow, bract-surrounded channel, called an ostiole, which has evolved to only allow the entrance of the fig’s species-specific agaonid wasps (Wiebes 1979; van Noort and Compton 1996). Inside, the female wasps pollinate the flowers as they oviposit their eggs. Their larvae then feed on the galls they have induced (Kjellberg et al. 2005). A few weeks later, the wasp offspring emerge simultaneously when the male flowers mature. After mating they leave their natal syconium to find a new receptive one. Since the pollinator lifespan is extremely short, usually only a few hours to two days (Kjellberg et al. 1988; Dunn et al. 2008; Wang et al. 2009), the newly emerged pollinator has to enter another available syconium within a short time period. Thus a continuous syconium crop presence would be necessary to sustain a local pollinator population.

However, variability in environmental conditions (e.g., low temperature, dry season, etc.), which decrease syconium production (Kjellberg et al. 1987; Spencer et al. 1996; Chen et al. 2004; Tzeng et al. 2006), also dramatically decrease pollinating wasp populations and can even result in a local extinction of these pollen carriers (Bronstein 1995; Harrison 2001; Bain 2012). While the cited studies have already established the environment – syconium production – pollinating wasp dynamics, none have described the trajectory of the pollinating wasp population in detail or explored the relationship between its recovery rate and the syconium phenology during seasonal conditions.

*Ficus microcarpa* is a widely distributed species planted worldwide. Its adaptability has allowed it to colonize various environments such as the American continent, the Hawaiian archipelago, and the South of Europe (McKey 1989; Kaufmann et al. 1991; Nadel et al. 1992; Doğanlar 2012). The associated pollinating wasp species of *F. microcarpa*, *Eupristina verticillata* Waterston has been observed in many places where *F. microcarpa* was introduced (Kaufmann et al. 1991; Beardsley 1998; Doğanlar 2012). The invasion of *F. microcarpa* seedlings indicates that the pollinators work efficiently. What are the characteristics of the tropical *F. microcarpa* that make it an efficient invasive species even in strongly seasonal areas such as North America? What are the phenological advantages that allow *F. microcarpa* to maintain its pollinating wasp population in its distribution area and in newly colonized lands?

In this study, we focus on a *Ficus microcarpa* population in Taipei, Taiwan where seasonality in syconium abundance was recorded (Hsieh 1992; Chen et al. 2004). The phenologies of both the mutualists, the syconium abundance and the pollinator population dynamics, were studied at the same time over a 14-month survey. The aim was to investigate the dual cycling of populations of figs/fig wasps to explore their adaptive strategy for seasonality.

## Methods

### Study species

*Ficus microcarpa* L.f. (subgenus *Urostigma*, section *Conosycea*) is a large evergreen, hemi-epiphytic monoecious tree with abundant aerial roots. Syconia are found axillary, singly or in pairs (Hill 1967; Tzeng 2004; Berg and Corner 2005). Ripe syconia measure about 6–10 mm in diameter, turning from reddish green to pink or purple, and are consumed by more than 200 frugivorous vertebrate species, mainly birds and some fruit bats (Shanahan et al. 2001). *Ficus microcarpa* is natively distributed from Southern and Eastern Asia to Northeastern Australia (Hill 1967; Chew 1989; Berg and Corner 2005). This species is also native to Taiwan where it is a common roadside tree in all urban areas. *Ficus microcarpa* is pollinated by the pollinating wasp *Eupristina verticillata* Waterston (Hymenoptera: Agaonidae *sensu* Cruaud et al. 2010) (Chen et al. 1999). Like many monoecious *Ficus* species, most *F. microcarpa* trees produce synchronous crops. However, some level of asynchrony within the same branch or among different parts of the tree crown has been reported in past studies (Hill 1967; McPherson 2005a; Lin et al. 2008; Yang 2011).

### Study site and climate

In order to investigate the typical *F. microcarpa* urban trees, 29 trees were surveyed along a 250 m stretch of road by the National Taiwan University campus in Taipei, Taiwan (25°00′43″N, 121°32′25″; 21 m above sea level). These trees are more than 25 years old, with heights ranging from 7 to 14 m.

**Figure 1:**
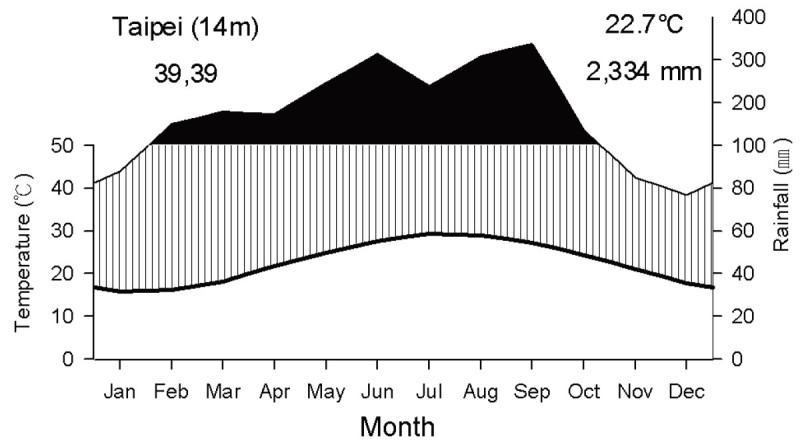
**Climate diagram of Taipei.** Mean monthly temperature, as given on the left axis, is plotted with the thick line. Mean monthly rainfall, as given on the right axis, is plotted with the thin line. The black area represents very moist periods and the shaded area, relatively moist periods. Meteorological data ranging from 1960 to 2009 were obtained from Taipei meteorological station in the Central Weather Bureau (http://www.cwb.gov.tw), located 3.7 km from sampling site.

Taipei has a humid subtropical climate. February to October is classified as a very moist period, while November to January is classified as a relatively moist period (Figure 1).The meteorological definition of seasons by the Central Weather Bureau (http://www.cwb.gov.tw) was adopted in this study: spring: March to May; summer: June to August; fall: September to November; and winter: December to February. The average temperature is 22.7°C, 29.4°C in summer and 18.1°C in winter (Taipei meteorological station in the Central Weather Bureau). Summers are humid and accompanied by occasional rainstorms and typhoons, while winters are short and mild.

### Syconium developmental stage classification

The development of syconia was classified into 5 stages according to Galil and Eisikowitch (1968). In the A phase, the syconium initiates and the female flowers grow inside. In the case of *F. microcarpa*, this period lasts about 0.5 to 1 week (Chen et al. 2004). The B phase is the female phase when female flowers mature and emit volatile compounds which attract pollinators (Hossaert-McKey et al. 1994; Gibernau et al. 1998; Grison-Pigé et al. 2002; Proffit et al. 2008). This is the only period during which pollinators can enter the syconia. The length of this period is typically 0.5 to 1 week, and can be extended to 2 weeks if pollinators do not enter the syconium and pollinate the flowers (Chen et al. 2004). In the C phase (interfloral phase), seeds and galls develop inside the syconia. This development takes 2 weeks in summer and fall, and 4 weeks in spring and winter. The D phase is the male phase: fig wasp offspring emerge while male flowers mature. Then female fig wasps loaded with pollen grains exit the syconia. In *F. microcarpa,* as in numerous other *Ficus* species, the D phase is short, lasting for a few hours to one day (Yang, unpublished data). During the following 1 to 3 days, the syconia drastically change in appearance from greenish pink at the wasps’ emergence to dark purple. This is the E phase where seeds mature and are ready to be dispersed. The purple syconia mainly attract birds in Taipei (Yang, pers. obs.). Although both D and E phases are mainly short, there exists developmental asynchrony among syconia of the same tree (Hill 1967; Lin et al., 2008; Yang 2011), and thus we can observe D or E-phase syconia on a tree for around 1 to 2 weeks.

### Phenological censuses

We conducted phenological censuses from May 2008 to July 2009, visiting each of the 29 trees at a weekly interval (the average was 8.2 days between censuses). During each survey, we cut down four branches from each tree; each branch was randomly selected from one of the four ordinal directions (North, South, East and West) of the tree crown. In this study, we used a 30–50 cm long branch located at a height of 2–5 m as our sampling unit. We recorded the number and developmental stage of the syconia in each sample. If we encountered late B or C phase syconia, we brought them back to the laboratory for further investigation of the foundress number.

During each survey, we also recorded the proportion of syconium-bearing branches on each tree. First, we divided the tree crown into 4 ordinal parts in similar locations where the sample branches were taken. Then, we estimated the proportion of syconium-bearing branches in each part. The estimations were categorized into 5 classes: 1= less than 25% of branches bearing syconium, 2= about 25% bearing syconium, 3= about 50%, 4= about 75%, and 5 = about 100%. We directly counted the syconium-bearing branches in all class 1 parts. We then used this data for the estimation of foundress population size as described in the following method. To estimate the total number of branches on the tree crown, we took numerous photographs of each tree on November 26, 2010. We carefully counted the number of branches from the digital images with image processing software (Adobe Photoshop CS3). In cases where it was difficult to take good quality photographs of some parts of the trees for a reliable count, we estimated the number of branches based on the average count of the other parts. We observed that the trees grew slowly and were trimmed once a year in spring and thus we assume that the variation in the number of branches during the survey period was small enough to be neglected.

### Foundress population monitoring

In this study, we regarded the syconia as natural bio-traps for pollinators. As such, we used the number of foundresses trapped in the B- or C-phase syconia as an indicator of the pollinating wasp population in the local environment. We considered this as a reliable indicator based on two assumptions. First, the number of foundresses in a given syconium is positively correlated with the current and local wasp cloud (i.e. the free-flying pollinators yet to enter a receptive syconium). However recent studies proved there is a density-dependent ostiole enclosure (Wang et al. 2009). When the foundress number is high, the ostiole closes more quickly to avoid too many foundresses. In this case, the foundress number might be saturated, and thus we tend to underestimate when there is an abundance of pollinator wasps. Second, although some foundresses could exit from a syconium after entering (Gibernau et al. 1996; Chen et al. 2001), the vast majority of the observed foundresses are positively correlated with the actual number of foundresses that had entered the syconia. The foundress number was previously used in several studies to reflect the effect of syconium seasonality (Bronstein and Hossaert-McKey 1996; Wang et al. 2005; Wang and Sun 2009). However, the weekly abundance, as presented in this study, was only seen in the study of Bronstein and Hossaert-McKey (1996).

We took the B and C phase syconia from the sample branches and cut them open to count the number of foundresses under a stereomicroscope. Although some foundresses might be mangled during the syconium development, they would not be decomposed, and thus were able to be pieced back together. We identified and recorded the phase of the sampled syconia. To ensure all potential pollinators had entered, only syconia in the late B phase or early C phase were analyzed. Foundresses in 8,073 syconia from 862 branches (28 trees, as one never fruited) were counted during the study period.

### Data analysis

A crop was defined as the succession of syconia from phases A to E for each tree. Sometimes trees can start a new crop before the previous crop was finished and is denoted as another overlapping crop. The period when an individual tree does not bear any syconia is called a flowering interval.

In this study, we also attempted to investigate the relationship between meteorological factors and syconium productivity. We considered three meteorological variables in this study: daily mean temperature, daily rainfall, and daily sunshine hours. We obtained these data from the Taipei meteorological station, located 3.7 km away from the study site. We then used the inter-survey average of the temperature and sunshine hour and inter-survey rainfall sum (for the first survey, we took a 7-day interval) for correlation tests. Principle component analysis (PCA) was used to identify and explore covariance patterns among meteorological factors. In the PCA, a correlation matrix was used. We tested the significance of each loading (eigenvector coefficient) by the bootstrap-confidence interval method (Peres-Neto et al. 2003).We performed a generalized linear model (GLM) with negative binomial error (R software, R Development Core Team 2009) to explore the correlation between syconium production in each phase and the principle components (PCs) of the meteorological data. Since the syconium developmental phases are progressive, we included the previous phase as a covariate for the B, C, D and E phase with a 1- or 2-week delay effect (depending on which one has higher correlation coefficient). In order to prevent the extremely high amount of rainfall brought by typhoon Sinlaku from influencing the results, the survey following the passage of typhoon Sinlaku (September 17^th^) was not incorporated in the analysis.

In order to represent the foundress dynamics in a different aspect, we developed four foundress indexes. Previous studies simply adopted “average foundress number,” which is the total number of foundresses divided by total sampled syconia (e.g. Bronstein and Hossaert-McKey 1996; Wang et al. 2005; Wang and Sun 2009) to reflect the foundress abundance. Here we separated the pollination frequency into two levels, the tree (individual) as well as the syconium level. In addition, the crowdedness of foundresses inside each occupied syconium should also be considered. Thus, we developed the following four indexes for each survey:

1.  Proportion of pollinated trees: the proportion of trees receiving pollinators among all receptive trees.
2.  The occupation rate: the proportion of syconia entered by foundresses among all dissected late B- ore early C-phase syconia on a given branch.
3.  Crowdedness index: the mean number of foundresses in occupied syconia on a given branch.
4.  Foundress population size: the approximate size of the pollinator cloud (the free-flying pollinators yet to enter a receptive syconia) of each survey, which was calculated by the number of foundresses sampled multiplied by the estimated number of total B-phase syconia:
  $$\mathrm{Foundress}\;\mathrm{population}\;\mathrm{size}=\sum_{j=1}^{29}\sum_{i=1}^{4}b_{\mathrm{ij}}f_{\mathrm{ij}}a_{\mathrm{ij}}$$

where *i* represents the ordinal parts (1–4) of the tree, and *j* represents the number of the tree (1–29). *b*_*ij*_ is the number of syconium-bearing branches at side *i* of tree *j*, *f*_*ij*_ is the number of syconia in the late B phase or early C phase, and *a*_*ij*_ is the average number of foundresses on the sampled branch, calculated by multiplying the occupation rate and the crowdedness index. In the instances when foundresses were not sampled from one to three sides of the tree crown, we used the average foundress number of all other sides on the same tree to represent *a*_*ij*_ for these sides. The number of syconium-bearing branches (*b*_*ij*_) for each tree direction, in reference to the 5 classes (see above) recorded in the field survey, is equal to either the actual counts of the branches in the case of class 1, or the product of the proportion of syconium-bearing branches and the total number of branches in the cases of classes 2–4.

## Results

### Phenology of syconium production

**Figure 2:**
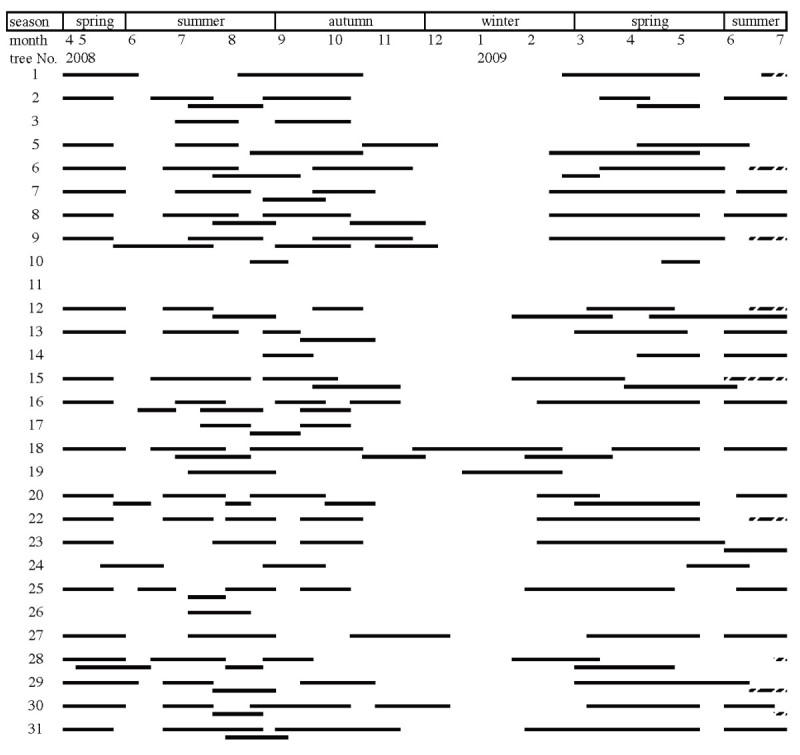
**Crop productions of 29** ***Ficus microcarpa*** **trees investigated in Taipei from April 29, 2008 to July 5, 2009.** Solid lines indicate complete crops. Dashed lines indicate unfinished crops.

**Figure 3:**
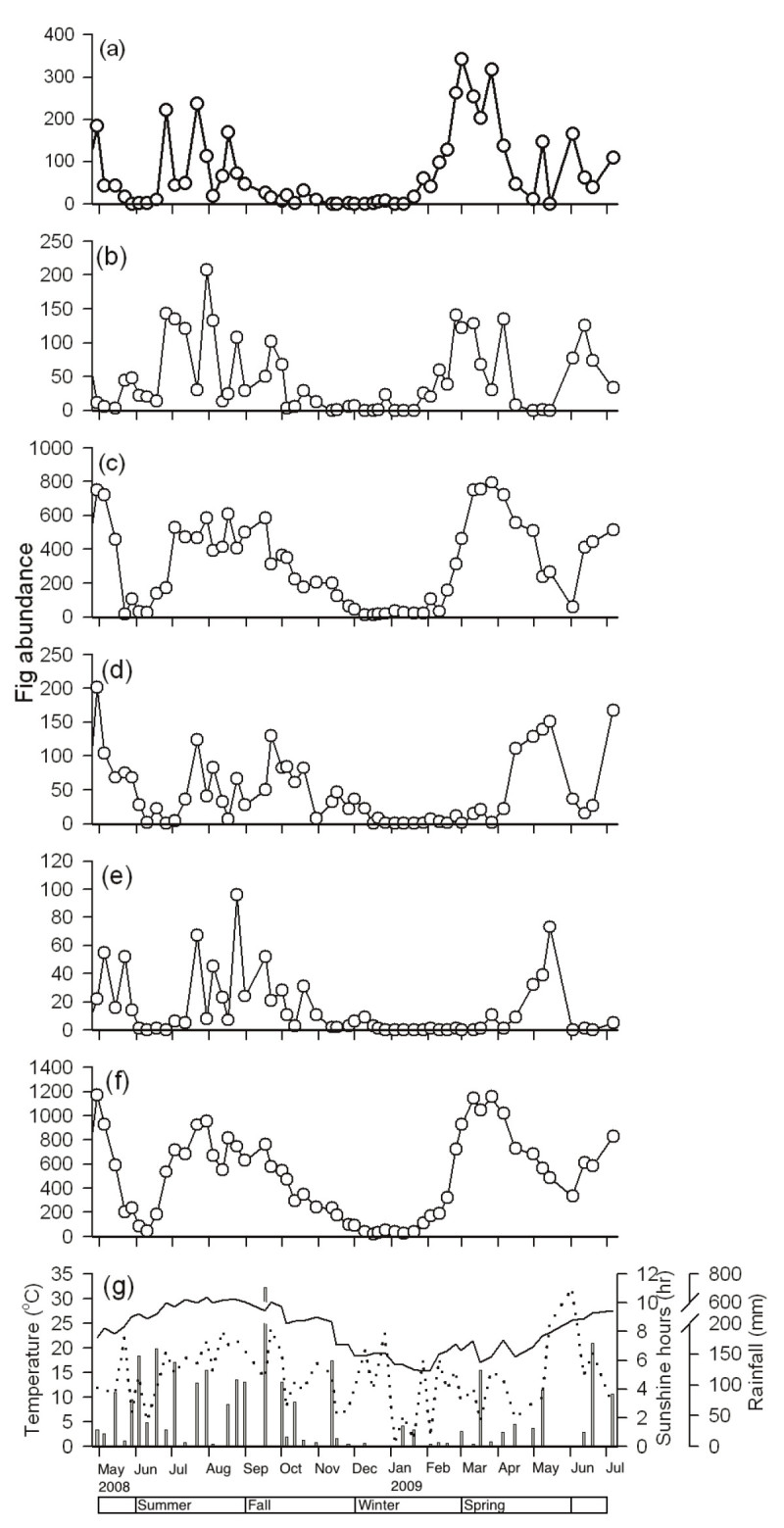
**Syconia abundance in each phase of** ***F. microcarpa*** **and the meteorological data in Taipei from April 28**^**th**^**, 2008 to July 5**^**th**^**, 2009: (a) A phase, (b) B phase, (c) C phase, (d) D phase, (e) E phase, (f) total syconia, and (g) meteorological data.** Solid lines represent temperature; dotted lines, sunshine hours; and bars, rainfall.

Syconia of *Ficus microcarpa* were present year-round, with obvious high yield and low yield seasons (Figure 2). A total of 158 crops were recorded from 29 fig trees. Individual trees produced 0–9 crops with an average of 5.4±2.7 (mean±SD) crops during the period of the 14-month survey, equivalent to 4.7 crops per year. There were two main syconium production periods: from July to October 2008 and mid-February to May 2009 (Figure 3f). Between them was a winter trough lasting two months. Thus, we classified the syconium phenology into three periods: the spring crop season, the summer-fall crop season, and the winter trough.

The spring crop season started with the increase in temperature when the winter ended (Figure 3a, f).The syconium development took 4 to 12 weeks (Figure 2). Nineteen of these trees were recorded as bearing syconia in the A or B phase over several successive surveys, which revealed a continuous production of new syconia for several weeks. This accounted for an immense number of A-phase syconia in this period (Figure 3a). Two peaks of D-phase syconia were observed: a small one at the end of March, and a larger one in mid-May. The trees then entered the flowering interval lasting 2 to 4 weeks (Figure 2). The summer crop season started in June (both 2008 and 2009). The syconium development was relatively shorter during this period and took about 4 weeks to finish a cycle. The syconium quantity decreased gradually in fall, and the last D-phase peak resulting from the end of 4 trees’ crops occurred in December. All except 2 trees stopped producing new syconia after November, and went into a long flowering interval lasting 2 to 3 months (Figure 2).

### Correlation with meteorological factors

The first principal component (PC1) explained 55.29% of the meteorological variability and was closely related to temperature (loading = 0.7054, *p*<0.01), while the second component (PC2), explaining 33.13% of the variance, was positively related to sunshine hours and was negatively related to rainfall (Table 1). In terms of the correlation between syconium abundance and meteorological factors, the syconium abundance of total counts and all phases except the A-phase were positively correlated with PC1 (all *p* s<0.05, Table 2). Furthermore, only E-phase syconia showed a positive correlation (*p*<0.05) with PC2.

**Table 1:** **Principal component analysis for the three meteorological factors**

Variables	PC1	PC2	PC3
Eigenvalue	1.6586	0.9938	0.3476
Explained variance of PC (%)	55.29	33.13	11.59
Meteorological factors			
Rainfall	0.4632	−0.7578**	−0.4595
Temperature	0.7054**	0.0014	0.7088**
Sunshine hours	0.5365	0.6525**	−0.5352

Asterisks flowing the loadings represent the significance level: *p<0.05, **p<0.01, ***p<0.001.

**Table 2:** **Correlation between meteorological factors and the abundance of syconia**+

Syconium phase	Covariance	PC1	PC2	PC3
A	None	0.0300	0.0905	−0.1493
B	A, 1-week delay	0.3211*	−0.0327	1.1363***
C	B, 2-week delay	0.3198***	−0.0684	−0.1052
D	C, 2-week delay	0.2110*	−0.0953	0.1024
E	D, 1-week delay	0.4988***	0.4667**	−0.01812
Total syconia	None	0.2110*	−0.0953	0.1024

Asterisks represent the significance level: *p<0.05, **p<0.01, ***p<0.001. + The variables used in the correlation was transformed by a principle component analysis.

### Pollinator abundance

**Figure 4:**
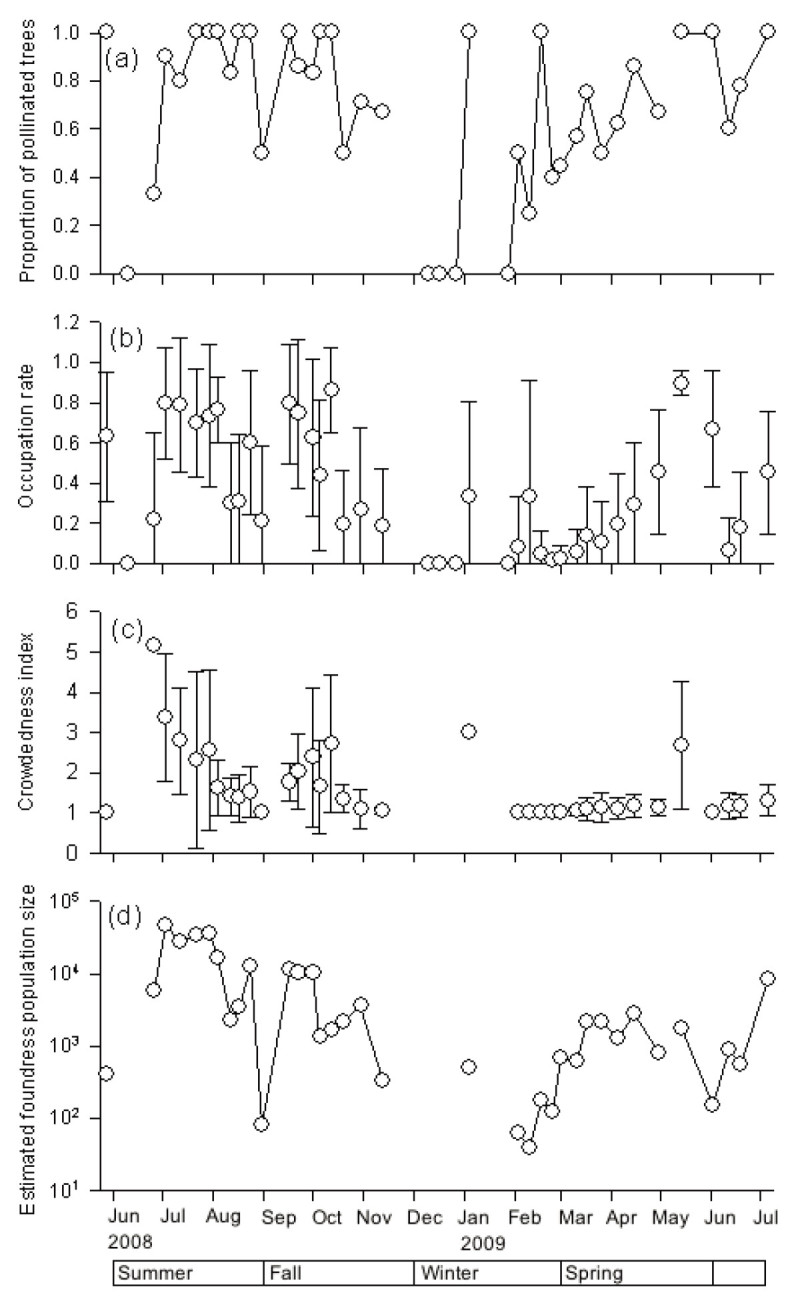
**Pollinator indexes of** ***F. microcarpa*** **in Taipei from May 27, 2008 to July 5, 2009: (a) Proportion of pollinated tree, (b) Occupation rate, (c) Crowdedness index, and (d) Estimated foundress population size in study area.** Vertical bars in **(b)** and **(c)** indicate standard deviation.

Among the 862 sampled branches from the different surveys, the occupation rate (i.e., the proportion of syconia occupied by pollinators) varied from 0 to 1, and the crowdedness index (number of foundress in the occupied syconia) varied from its minimum of 1 up to 9.2. The estimated foundress population size ranged from 0 to 45,957. All the foundress indexes varied greatly during the survey (Figure 4).

During the spring crop season, the proportion of pollinated trees increased steadily (Figure 4a), and the occupation rate increased from 0–0.4 in February to 0.9 in May (Figure 4b), with an average of 0.2. However, the crowdedness index remained at an average of 1.2 (Figure 4c). These indicated that the number of receiving syconia was greater than the available pollinators. The occupation rate, average foundress number, and estimated foundress population size decreased in June 2008 and 2009 during the flowering intervals of numerous trees. However, all the indexes recovered to a higher value in July in both 2009 and 2008 (Figure 4).

The weekly pollinator indexes were highest from July to October (Figure 4). The average occupation rate was 0.6 and ranged from 0.2 to 0.9 (Figure 4b). The crowdedness index was 2.0 on average. The estimated foundress population size was greater than 30,000 in July 2008 (Figure 4d). Although there was a drastic decline in August, occupation rates recovered in the following month, but the crowdedness indexes dropped afterwards (Figure 4c). The estimated foundress population size kept decreasing from fall to winter, indicating a decline in pollinator population during this period.

The four pollinator indexes were low during the syconia production trough. The production of new syconia was intermittent from December to February and thus resulted in a lack of data in some weeks. Based on available data, occupation rates during this period were low (Figure 4c). Only one tree (Number 19) that grew new syconia in January was pollinated successfully. Estimated foundress population size was less than 200 during this period.

**Figure 5:**
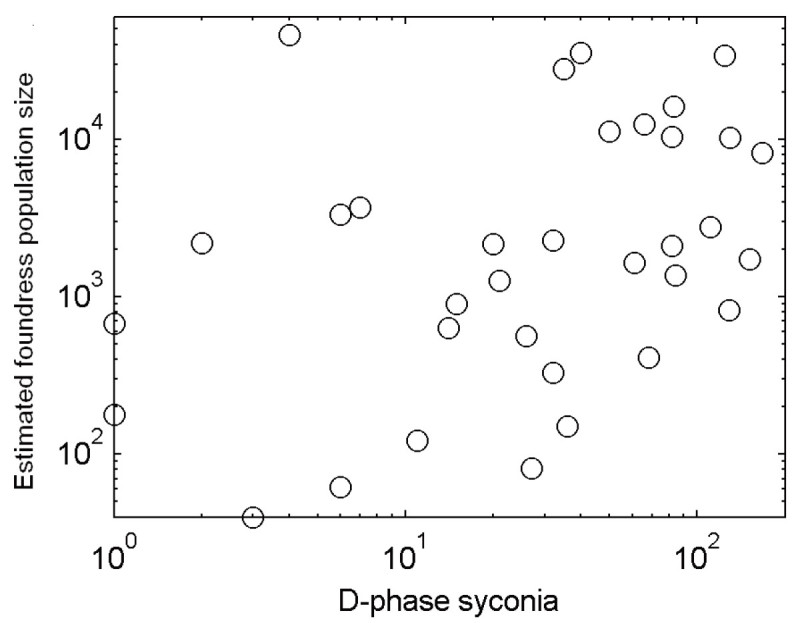
**A plot of estimated foundress population size versus D-phase syconia quantity.** The correlation between these two variables was significant (*p*<0.05). There were two weeks in which no D-phase syconia were censused but foundress population size was not zero. These took place on July 25, 2008 and January 3, 2009 respectively.

Estimated foundress population size was positively correlated with D-phase syconia abundance (Pearson correlation test, both variables were log transformed, *p*<0.001, *R*^2^ = 0.45, Figure 5). Note that there were two weeks where no D-phase syconia were observed but the foundress population sizes were not zero (Figure 4d). These two weeks revealed pollinator immigration events, which happened at the start of the summer-fall flowering season (July 25, 2008), and also in the winter trough (January 3, 2009).

## Discussion

Our study showed the dual dynamics of syconium abundance and the pollinating fig wasp population and established the correlation between them. The phenology of *Ficus microcarpa* in Taipei tracked seasonal variations, which can be separated into the spring flowering season, the summer-fall flowering season, and winter trough. The corresponding pollinating wasp population expanded in spring, maximized in summer and then shrank to a very low level in winter.

We found that PC1, which showed a significant high loading on temperature, was the most significantly correlated factor with syconium abundance, suggesting that this is an important factor determining the phenology of *F. microcarpa* in Taipei. A higher syconim yield in warm seasons was also reported in several other studies of *F. microcarpa* in other subtropical areas, as summarized in Table 3. In this study, the B, C, D, and E phases were positively linked with temperature and also with pollinating wasp activity. Numerous studies showed the positive effects of temperature on insect activity (Willmer 1983,1985; Kalin Arroyo et al. 1985), which is congruent with the number of C-phase syconia, which are the consequence of pollination. Also, temperature has a clear effect on Hymenoptera larval development (Ando et al. 2006; Dhillon and Sharma 2009) and seed maturation (Bronstein and Patel 1992; Tzeng et al. 2005; Ellis 2011), which are the processes involved in the D and E phases. In addition, PC2, which is positively linked to sunshine hours and negatively to rainfall, was statistically correlated with the abundance of E-phase syconia, the seed dispersal phase. The effect of light has been documented to be an important factor for the ripening of a wide range of plant species (Wright and van Schaik 1994; Zimmerman et al. 2007). Thus, the *Ficus* species do not deviate from the general pattern of fruit ripening.

**Table 3:** **Phenology studies on** ***Ficus microcarpa***

Study site	Study year	Duration (mo)/ interval (day)	Number oftrees	Phenology	Crops produced per tree per year	Crop length	Reference
Hong Kong	Jan-Nov 1964	11/14-30	20	Continuous production by species as a whole and by many individuals. All trees had a large crop in spring. 90% had a second crop in summer.	1-5, most 3-4	1-4 months	Hill 1967
Singapore	Oct. 1982-Feb 1984	17/7-17	8	No clear pattern.	2-6, mean 4.4	30 days	Corlett 1984
Taipei (Taiwan)	Jan. 1991- Feb. 1992	14/7-10	84	Continuous production with main peaks in Apr.-Jan and Jul.-Sep.	0-4, most 2-3	26 days in June	Hsieh 1992
Sao Carlos (Brazil)	Mar. 1991- Feb. 1992	12/15	19	Continuous production; syconia and leaf production were sequentially related.	2.26±0.81(meant±SD)	110.75 days	Figueiredo et al. 1995
Taipei (Taiwan)	Aug. 1992- Nov. 1998	76/7	35	2 gaps: late Apr. to early May, lasting 3 weeks; late Oct. to Feb lasting 5.8 weeks. Positive correlation with temperature but not rainfall.	2.1, with 25% aborted	5.97-10.28 weeks	Chen et al. 2004
Brisbane (Augstralia)	Sep. 1997- Aug. 2001	48/30	8	Female phase: Late spring peak; Male phase: more present in warmer months.	No records	No records	McPherson 2005a
Guangzhou (China)	Mar. 2005- Sep. 2006	19/1/7-14	10	Higher proportion of each phase from Aug. to Sep. and Nov. to Mar. A, B, C phase is negatively correlated with temperature	1-4, most 3-4	1-2 months	Lin et al. 2008

The substantial crops in spring provide favorable conditions for their pollinating agents to expand after the drastic population drop in winter. Considering the high reproductive potential of the pollinating wasps (20–80 offspring per foundress, Chen 2001), the pollinator population has the ability to grow exponentially. Our estimated foundress population size coincides with the theoretical logistic growth pattern of a population, with exponential growth in spring and the saturation period in summer (Figure 3e).

The abundance of syconia in spring was also recorded in previous studies in Taipei (Hsieh 1992; Chen et al. 2004), as well as in Hong Kong (Hill 1967) and in Brisbane (McPherson 2005a) (Table 3). In fact, the simultaneous production among trees might be a general physiological response of fig trees after harsh conditions, since it has been reported after extreme climatic events, such as tropical cyclones (Bronstein 1995; Bain 2012) and ENSO-induced droughts (Harrison 2001), and the occasional unfavorable season (Kjellberg et al. 1987; Tzeng et al. 2006; Spencer et al. 1996). After a period of low syconium density, pollinating wasps are rare. The immense crops following harsh conditions can be seen as an investment from the trees. The bigger the crop is, the greater the chance to attract a pollinating wasp. Once these trees were visited, the first-generation offspring of pollinators have a high likelihood of pollinating other syconia (number of receptive syconia is greater than the number of pollinators) with a weaker pollen competition (the crowdedness index remained at 1.2). Thus, the benefits from their male function, pollen production, may offset the cost of being unpollinated.

Compared to the spring crops, the summer-fall crops may benefit more from their female function, seed production. Previous studies of *F. microcarpa* reported an abundance of seed production two times greater in the summer-fall season than in the spring season. For example, the proportion of seeds per syconia was 23.8% during the summer-fall but only 9.7% during spring seasons respectively at Taipei (Chen 2001); the average number of seeds was 10.63 and 28.77 in June and September, respectively, but only 4.09 and 5.15 in March and December, respectively at Guangzhou, China (Lin et al. 2008). Furthermore, a higher temperature shortens the duration of syconium development (Bronstein and Patel 1992; Chen et al. 2004; Tzeng et al. 2005; this study) and enables more crops, allowing even more seeds to be produced.

Our study also revealed another adaptive strategy for maintaining the fig/fig wasp mutualistic relationship. Several studies focused on the sustainability of the pollinator population in winter, approaching from the perspective of the minimum required population size (Bronstein et al. 1990; Anstett et al. 1995,1997). Here in Taipei with distinct seasonal changes, we showed that a small number of pollinating wasps could spark the whole summer population. In Taipei, *Ficus microcarpa* succeeds in reserving the pollinators by bearing syconia on a few trees (Hsieh 1992; this study) and sheltering the pollinator larvae in C-phase syconia during the cold and rainy seasons. In other locations where *F. microcarpa* trees cannot bear syconia in winter due to environmental conditions, the wasp population might be sparked by the migrants, which is not a problem since the tiny agaonid wasps are excellent gliders (Compton et al. 2000 Harrison and Rasplus 2006) that can travel more than 100 km (Ahmed et al. 2009). Thus, an occasional local extinction and wasp irruption will occur, and the reproduction of *F. microcarpa* can still be assured in some seasons, as was the case reported in Brisbane (McPherson 2005b) where seeds in *F. microcarpa* syconia were found in May to July but not in August (the last month in winter).

*Ficus microcarpa* has been documented as an invasive species in many places where it was initially planted for ornamental use (McKey 1989; Nadel et al. 1992; Beardsley 1998; Doğanlar 2012). The data presented in this study uncovers at least three advantageous characteristics for adapting in an invasive condition. First, the pollinating wasps can greatly increase their population from one single crop. Second, they do not seem to enter syconia when other female wasps are inside but instead migrate to other available syconia, thus maximizing the reproductive outcome. This was supported by the crowdedness index which did not follow the increasing trends of occupation rate or fig tree proportion loaded with pollinators. Finally, the pollinating wasps for *Ficus mircrocarpa* must be good travelers. Therefore, with the above advantageous characteristics and through a dual cycling fluctuation in syconium phenology and wasp population dynamics, *F. microcarpa* has become an adaptive and invasive fig species worldwide.

## Conclusion

The present study showed a dual cycling in phenological patterns of fig production and wasp population dynamics to adapt to seasonal climate changes in northern Taiwan. Responding to the seasonal changes in climate, *Ficus microcarpa* at Taipei exhibited three seasons in its annual phenology: the spring crop, summer-fall crop and winter trough seasons. With the small amount of pollinators from the winter syconia of the same fig population and potential immigrated foundresses from other populations, the pollinator population size can increase very quickly from almost zero to over 40,000 wasps for this 29-tree local population within a year. Some advantageous characteristics have pre-adapted *F. microcarpa* into a good invasive fig species around the world.

## Authors’ original submitted files for images

Below are the links to the authors’ original submitted files for images. Authors’ original file for figure 1 Authors’ original file for figure 2 Authors’ original file for figure 3 Authors’ original file for figure 4 Authors’ original file for figure 5
