# Supplementary figures and images for: Phenology and pollinating wasp dynamics of Ficus microcarpa L.f.: adaptation to seasonality
Source: Bot Stud. 2013 Aug 21;54:11. doi: 10.1186/1999-3110-54-11 (PMC5430316; doi:10.1186/1999-3110-54-11)

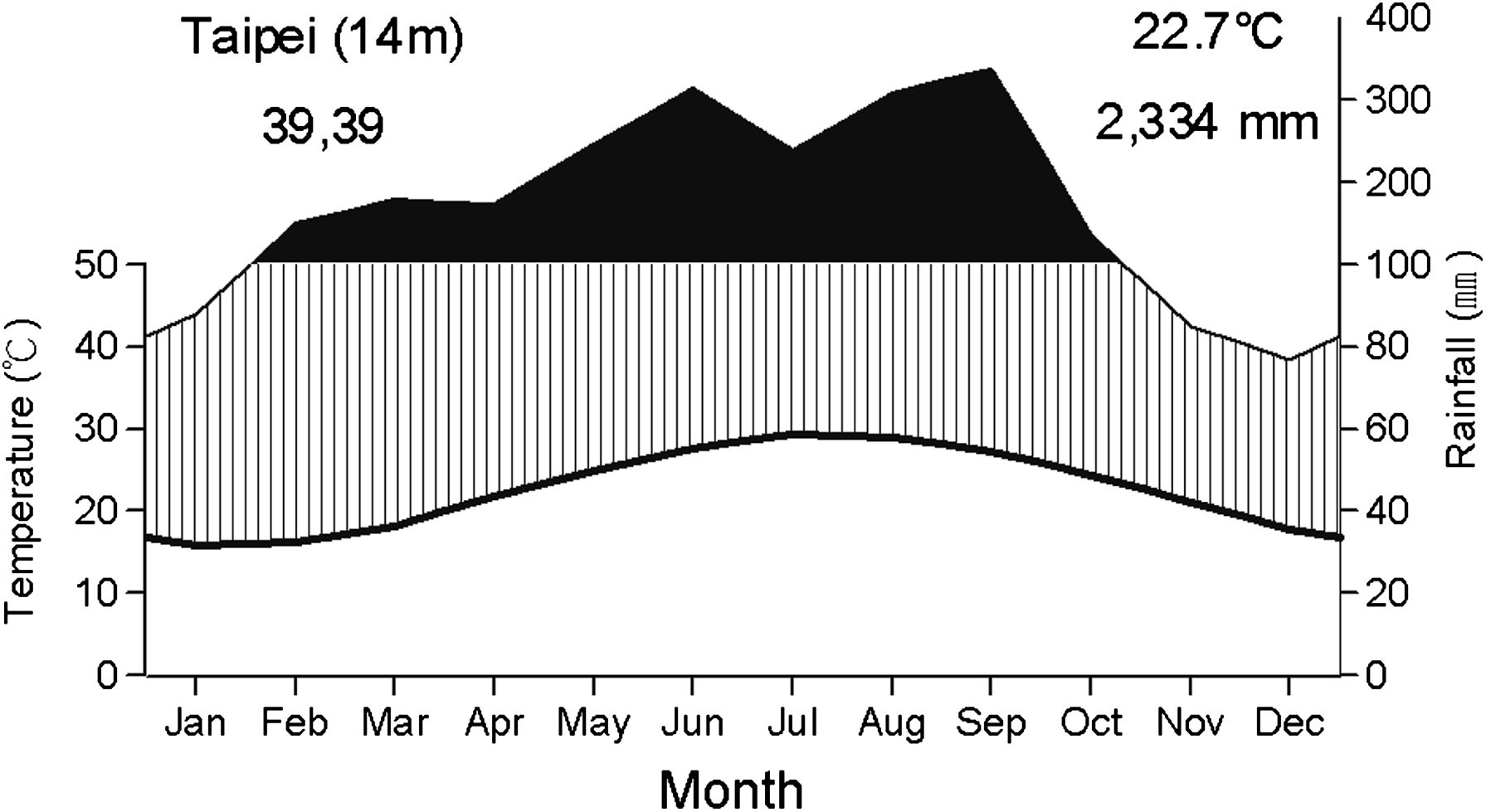

Supplement: Supplementary file 1 — Authors’ original file for figure 1 [file 40529_2012_11_MOESM1_ESM.tif]

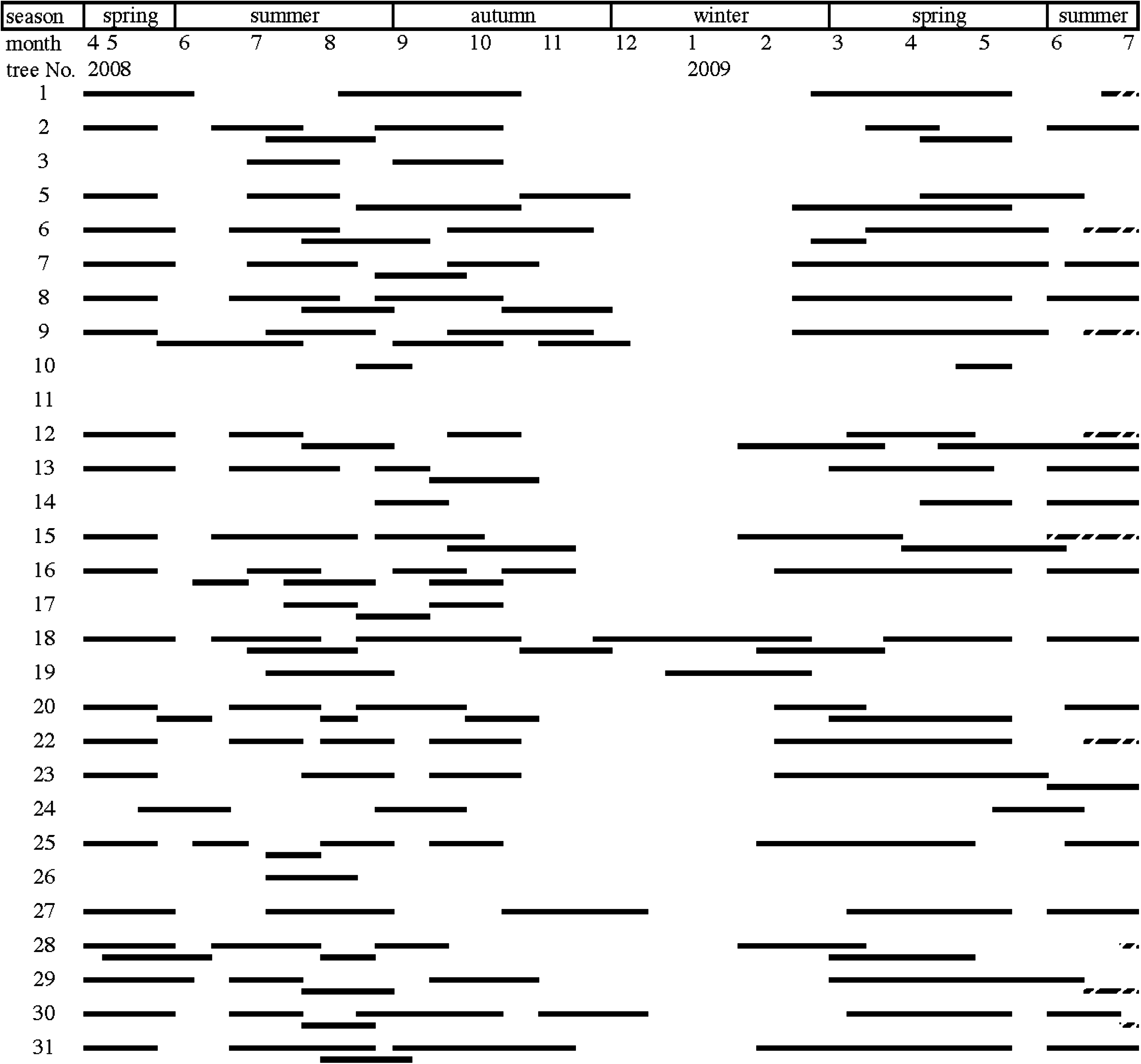

Supplement: Supplementary file 2 — Authors’ original file for figure 2 [file 40529_2012_11_MOESM2_ESM.tif]

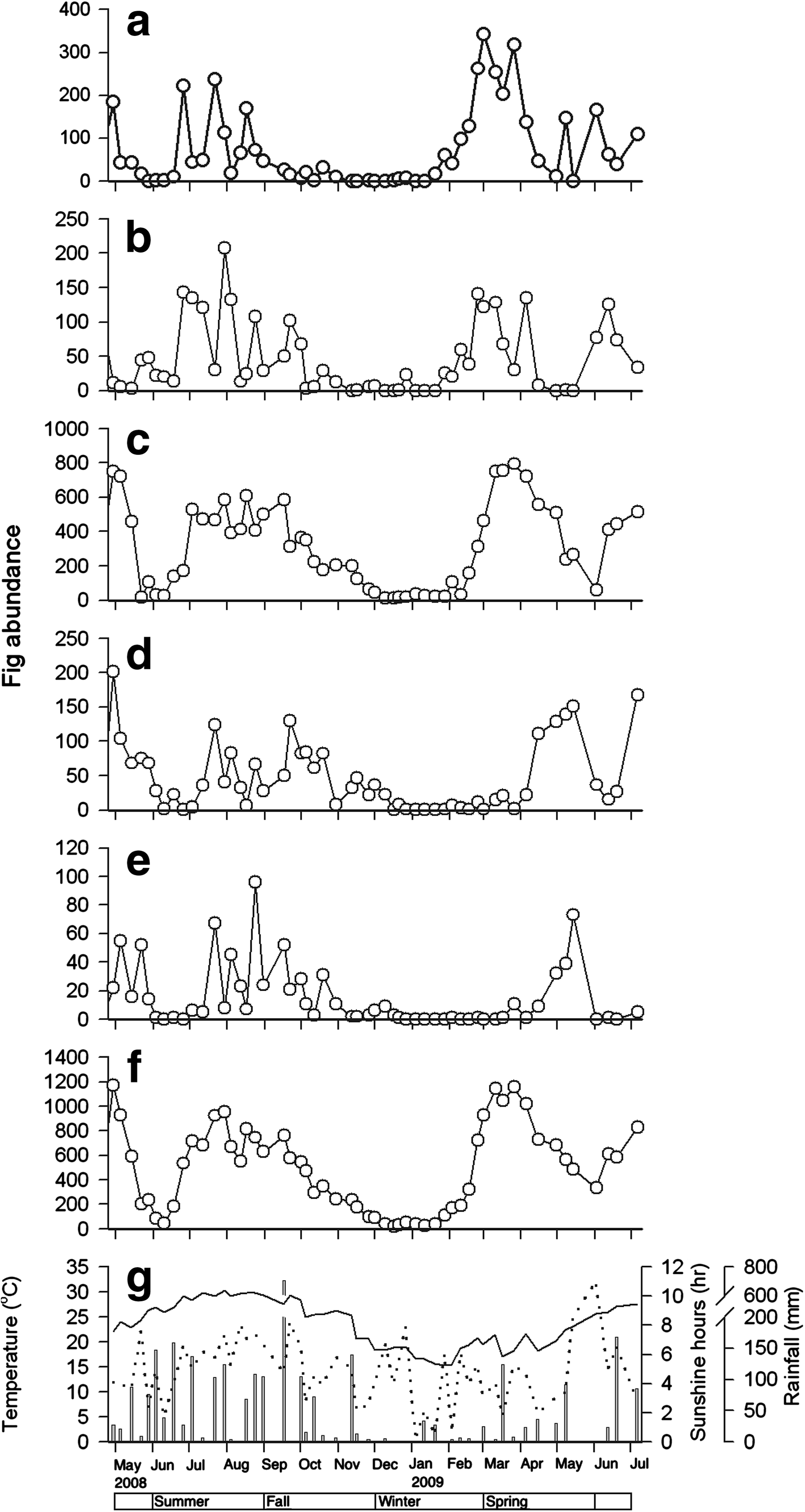

Supplement: Supplementary file 3 — Authors’ original file for figure 3 [file 40529_2012_11_MOESM3_ESM.tif]

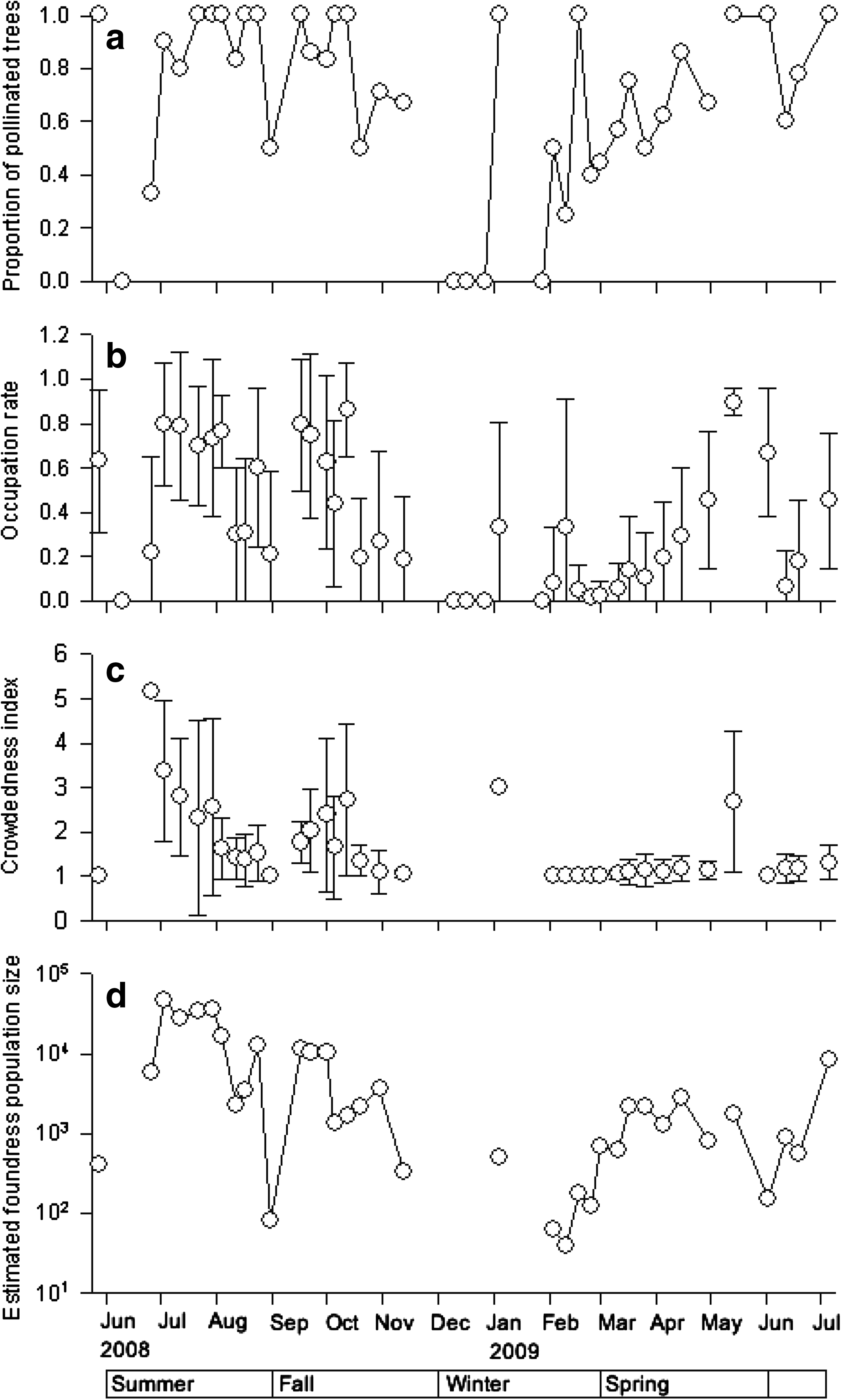

Supplement: Supplementary file 4 — Authors’ original file for figure 4 [file 40529_2012_11_MOESM4_ESM.tif]

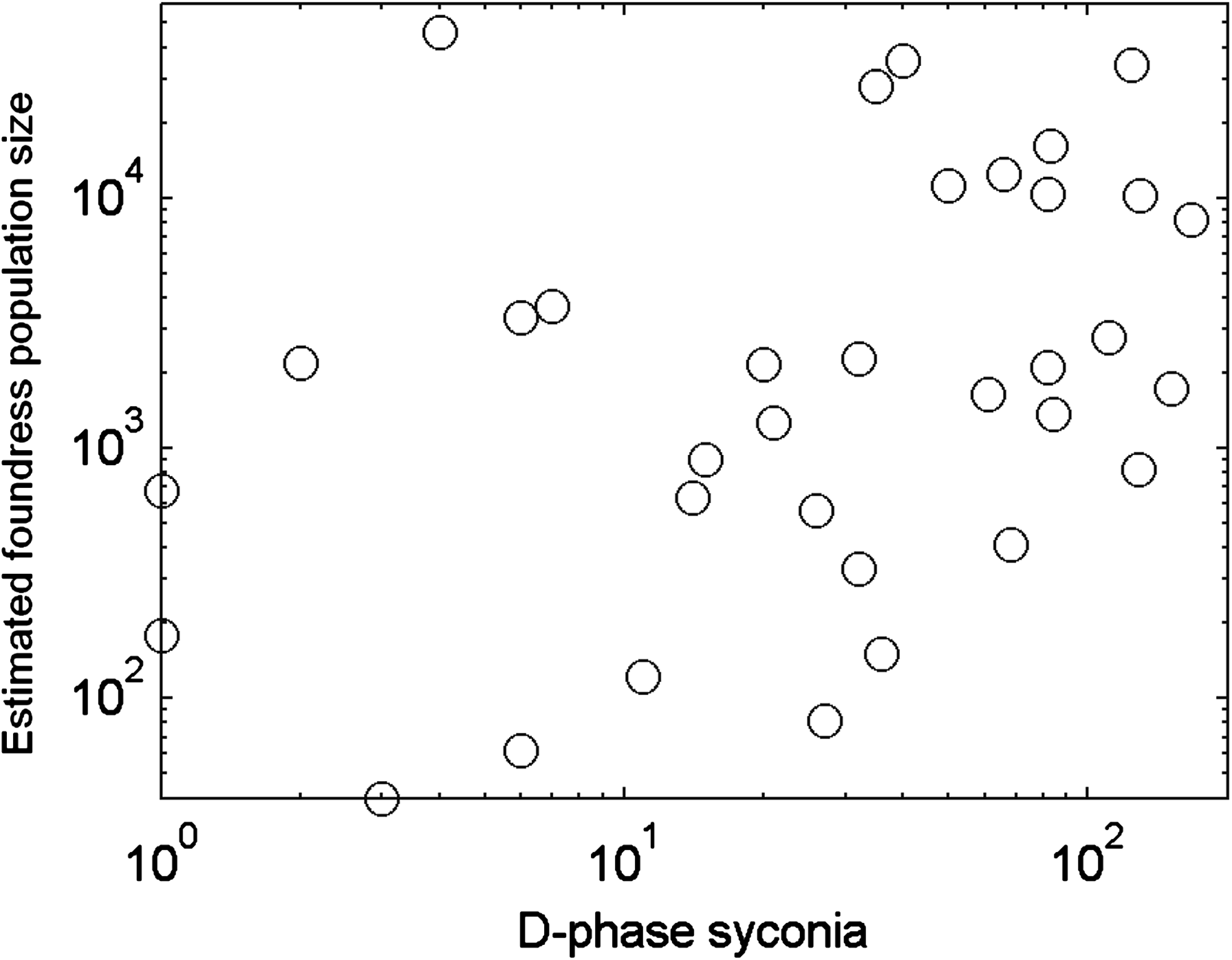

Supplement: Supplementary file 5 — Authors’ original file for figure 5 [file 40529_2012_11_MOESM5_ESM.tif]
